# The prognostic utility of pre‐treatment neutrophil‐to‐lymphocyte‐ratio (NLR) in colorectal cancer: A systematic review and meta‐analysis

**DOI:** 10.1002/cam4.4143

**Published:** 2021-07-26

**Authors:** Mate Naszai, Alina Kurjan, Timothy S. Maughan

**Affiliations:** ^1^ Medical Sciences Division University of Oxford Oxford UK; ^2^ Nuffield Department of Orthopaedics, Rheumatology and Musculoskeletal Sciences Botnar Research Centre University of Oxford Oxford UK; ^3^ MRC Oxford Institute for Radiation Oncology University of Oxford Oxford UK

**Keywords:** colorectal cancer, neutrophil‐to‐lymphocyte ratio, NLR, prognosis

## Abstract

**Background:**

Inflammation is a hallmark of cancer, and systemic markers of inflammation are increasingly recognised as negative prognostic factors for clinical outcome. Neutrophil‐to‐lymphocyte ratio (NLR) is readily available from routine blood testing of patients diagnosed with cancer.

**Methods:**

Peer‐reviewed publications from PubMed/MEDLINE, Web of Science and EMBASE were identified according to the Preferred Reporting Items for Systematic Reviews and Meta‐Analysis (PRISMA) guidelines. Hazard ratios (HR) for overall survival (OS) and surrogate endpoints (SE; comprising disease‐, recurrence‐ and progression‐free survival) were pooled using a random effects model. Additional analysis was carried out to further investigate NLR as an independent prognostic factor and account for heterogeneity.

**Results:**

Seventy‐one eligible papers comprising 32,788 patients were identified. High NLR was associated with poor clinical outcomes. Significant publication bias was observed, and larger studies also adjusted for more covariates. Correcting for publication bias in multivariate studies brought our best estimate for true effect size to HR = 1.57 (95% CI 1.39–1.78; *p* < 0.0001) for OS and to HR = 1.38 (95% CI 1.16–1.64; *p* = 0.0003) for SE.

**Conclusions:**

NLR is confirmed as an easily available prognostic biomarker in colorectal cancer, despite the limitations of some studies previously reporting this finding. As such, it should be routinely collected in prospective clinical trials. While more standardised and rigorous large‐scale studies are needed before high NLR can be fully assessed as an independent predictor of CRC progression and outcome, the data suggest that it may be used to highlight individuals with tumour‐promoting inflammatory context.

## Graphical abstract

Systemic inflammation is increasingly recognised as a key determinant of cancer survival. Consequently, readouts of inflammatory status have received immense research focus lately as potential prognostic factors. In this report, the authors explore a simple full‐blood‐count‐derived metric, the neutrophil‐to‐lymphocyte ratio (NLR), as a prognostic factor in colorectal cancer. Based on 71 publications, accounting for 32,788 patients, high NLR emerges as a readily available predictor of poor clinical outcome both for overall survival and surrogate endpoints, such as disease or progression‐free survival.

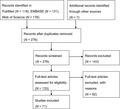

## 1 INTRODUCTION

Colorectal cancer (CRC) is the third most prevalent cancer globally.^1^ Despite the considerable improvement of survival rates in the past five decades,^2^, ^3^ CRC remains the second most common cause of cancer mortality, contributing close to a million deaths annually worldwide.^4^ While average survival ranges between 60% and 70%, the actual outcome varies greatly: from 90% 5‐year survival in early‐stage, localised colon cancer to just 14% in CRC with distant metastases.^3^ Prognostic factors can influence clinical decision making, and improve wellbeing by better aligning patient needs with available care.^5^ Additionally, through focused research, what is first identified as a prognostic factor may ultimately lead to the development of novel treatment strategies.^6^

Risk stratification strategies are currently guided by patient characteristics (e.g. age,^7^ sex^8^) and tumour‐specific features.^9^, ^10^ The European Society for Medical Oncology (ESMO) highlights the Eastern Cooperative Oncology Group (ECOG) performance status and the presence of comorbidities as relevant patient‐level prognostic traits. On the other hand, TNM stage, mismatch repair, microsatellite instability, invasion status and carcinoembryonic antigen (CEA) levels are established tumour‐specific prognostic factors.^11^, ^12^ In addition, it is increasingly recognised that inflammation and immune cells play an important role in tumorigenesis,^13^ therefore several inflammatory markers are being extensively investigated for their prognostic and predictive values. For example the modified Glasgow Prognostic Score (mGPS), which combines plasma albumin and C‐reactive protein levels, reflects systemic inflammatory status and has shown potential as a useful tool in CRC prognosis.^14^ Moreover, the Immunoscore assay that assesses the tumour immune infiltrate^15^ has recently been endorsed by ESMO.^11^

Ratios of full blood count (FBC) components (e.g. platelet‐to‐lymphocyte ratio (PLR), lymphocyte‐to‐monocyte ratio (LMR)) have recently joined the ranks of potential prognostic factors. Unlike the Immunoscore, these metrics are simple, cheap, widely available and non‐proprietary, holding the potential to provide an insight into the immune status of the patient.^16^, ^17^, ^18^, ^19^, ^20^ Of these ratios, the neutrophil‐to‐lymphocyte ratio (NLR) emerged as a prime surrogate readout of immune status for several reasons. First, in the context of tumour immunity, lymphocytes are best known for their anti‐tumour role; therefore, low lymphocyte counts may indicate poor cell‐mediated immunity.^21^ On the other hand, neutrophils—the most abundant immune cell type in circulation—are also often found to be recruited to tumours.^22^, ^23^ Like lymphocytes and macrophages, they play an active, reciprocal role in the context of cancer: tumours can induce elevated production of neutrophils in the bone marrow as well as recruit them to the site,^24^, ^25^ where they will then be polarised towards pro‐tumour and metastasis‐promoting phenotypes through TGFβ‐ and G‐CSF‐dependent mechanisms.^26^, ^27^, ^28^, ^29^ Therefore, elevated numbers of circulating neutrophils may be linked to tumour‐promoting inflammation. Overall, this makes NLR a metric that encapsulates both tumour‐promoting and anti‐tumour immunity, and therefore can potentially offer prognostic or even predictive value in CRC.

Here, we present the results of a systematic review and meta‐analysis that sought to assess the prognostic utility of pre‐treatment blood NLR in CRC and metastatic CRC (mCRC) for overall survival (OS) and progression‐, recurrence‐, or disease‐free survival (henceforth collectively termed surrogate endpoint (SE)). While several systematic reviews have already explored the prognostic value of NLR, the latest studies included were published in the year 2016.^30^, ^31^, ^32^, ^33^ Since then, however, the field has seen a marked increase in publications that offer valuable data about NLR in CRC. Combined with our non‐restrictive inclusion criteria, this enabled our work to capture more than 50 new studies, allowing for a more accurate estimation of true effect size, detailed subgroup analyses and meta‐regressions, for which previous studies were not adequately powered.

## 2 METHODS

The systematic review and meta‐analysis were conducted according to Preferred Reporting Items for Systematic Reviews and Meta‐Analysis (PRISMA) guidelines. The review protocol was registered with the PROSPERO registry (CRD42020176389) prior to the beginning of work.

### 2.1 Paper search protocol

Papers from PubMed (1946–2020), Web of Science (1945–2020) and EMBASE (1974–2020) were searched for peer‐reviewed primary research publications using the following search terms: “(hazard ratio) AND (survival OR mortality) AND (neutrophil lymphocyte ratio OR neutrophil‐lymphocyte ratio OR neutrophil‐lymphocyte OR neutrophil‐lymphocyte‐ratio OR NLR) AND (colon OR bowel OR colorectal OR rectal) AND (cancer OR carcinoma OR tumour OR tumor OR adenoma OR neoplasm OR malignancy) NOT (systematic review OR systematic‐review OR meta‐analysis)”. Additional papers were identified by screening bibliographies of included publications.

The selected studies were imported into Covidence software, which removed duplicates and allowed screening to be carried out in three separate stages. All titles, abstracts and full texts of selected articles were screened independently by the two reviewers (MN and AK). Consensus decision was made for any disagreements.

### 2.2 Study selection

#### 2.2.1 Inclusion criteria

We included all full‐text, peer‐reviewed, prospective or retrospective studies that reported HR and 95% CI of subsequent events (e.g. OS, disease‐, progression‐ or recurrence‐free survival) in patients with early‐stage CRC (American Joint Committee on Cancer (AJCC) stages I–III) and mCRC (stage IV) relative to pre‐intervention blood NLR levels.

#### 2.2.2 Exclusion criteria

Any conference abstracts and posters were excluded from the analysis (*N* = 33). Publications that did not report on CRC or mCRC (*N* = 3), NLR‐specific HR or the corresponding 95% CI (*N* = 10), or a specific NLR cut‐off (*N* = 5) were also excluded. Additionally, we did not include publications that were not written in English (*N* = 3) or studies where NLR was not sourced from blood (*N* = 1). Papers that reported on patients with an emergency presentation of CRC were also excluded (*N* = 1) to avoid spurious findings due to the acute inflammatory environment that is associated with an emergency presentation. Finally, in cases of studies with overlapping data (using same patient cohorts), the older study was always excluded (*N* = 5).

Reasonable attempts were made to request unreported HR or 95% CI data from the authors. As a result, one additional study^34^ was included in the analysis with this information provided.

### 2.3 Data extraction

The following data were extracted for each paper: name of the first author, publication year, univariate and/or multivariate HR and 95% CI for OS and/or SE, time period of patient recruitment, country of patient recruitment, cancer type (colon or rectal cancer, CRC, mCRC), tumour stage, median or mean age of participants, number of participants, number of male participants, length of patient follow‐up, means of determining NLR cut‐off, NLR cut‐off value and covariates adjusted for in multivariate analysis (e.g. sex, age). Progression‐, recurrence‐, or disease‐free survival as well as time to remission were coded individually as distinct endpoints but also combined into a single outcome measure collectively referred to as SE to capture all subsequent events regardless of cancer stage and maintain adequate power.

Data were extracted manually and independently: each paper was scanned by MN or AK, and relevant info was exported into Microsoft Excel (Version 2007) software for data organisation.

### 2.4 Risk of bias assessment of primary studies

The quality of primary studies was assessed using the Newcastle–Ottawa Quality Assessment Scale (NOS) for cohort studies.^35^ The score is assigned based on the individual study's quality of reporting of cohort selection, comparability and outcome. Studies with NOS score of ≥6 were considered to be high‐quality. The assessment was carried out independently by AK and MN, and any disagreements were resolved by joint discussion.

### 2.5 Statistical analysis

Data analysis was carried out in RStudio (Version 1.3.1073^36^) using the meta,^37^ metaphor^38^ and dmetar^39^ packages. Published hazard ratio data were converted into natural logarithms (logHR) for use in generic inverse variance random effects model to pool effect size estimates of the HR.

Between‐study heterogeneity was evaluated using the Higgins & Thompson's *I^2^* statistic^40^—a measure less sensitive to the number of studies analysed than the commonly used Cochran's *Q*.^41^

In the multivariate datasets, meta‐regression analyses were carried out for continuous variables, whereas subgroup analyses were performed for categorical variables. For subgroup analyses, we looked both at study‐specific characteristics (e.g. country of study, proportion of patients with metastasis (AJCC stage), specific secondary outcome measures grouped under SE) and the common covariates that the studies adjusted for (e.g. age, sex, FBC‐derived inflammatory markers other than NLR). See Table S1 for the full list of considered variables. Groups were compared if there were at least four studies in each subgroup. Random effects models were used within groups. Between‐groups comparisons were performed using fixed effects models (also referred to as mixed‐effects models), with the exception of the study's countries, which were analysed using a more appropriate random effects model.

### 2.6 Publication bias

Publication bias was assessed using funnel plots in which study estimates (log(HR)) on the *X*‐axis are plotted against their standard error (precision) on the *Y*‐axis.^42^ In the absence of bias, the plot appears symmetric, resembling a funnel. If small‐study biases are present, the plot points will be skewed towards the right, with smaller, less precise studies reporting higher effect sizes. Egger's test of the intercept^43^ was used to assess the significance of funnel plot asymmetry. Small‐study biases were then corrected by Duval & Tweedie's trim‐and‐fill method.^44^

## 3 RESULTS

**FIGURE 1:**
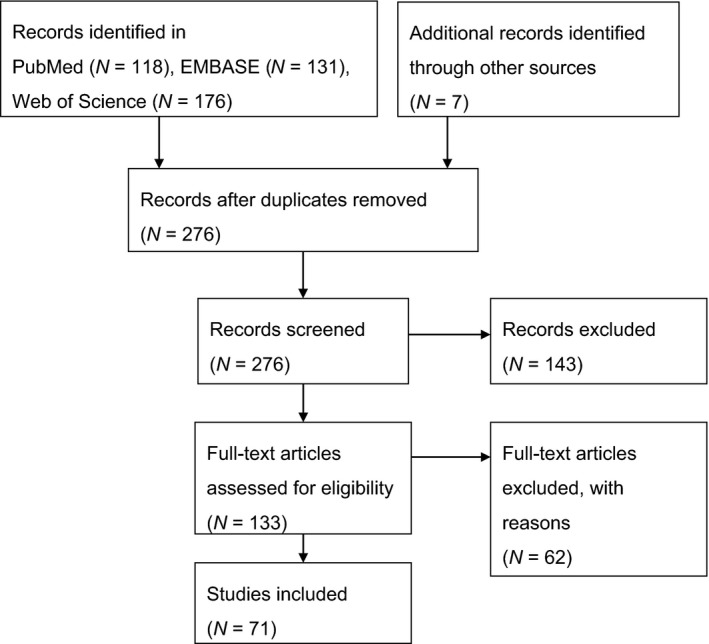
PRISMA flow diagram summarising systematic review study selection. A total of 425 records were retrieved through our search of PubMed/MEDLINE, EMBASE and the Web of Science. An additional 7 studies were identified by screening the bibliography of included studies. After removing duplicates and an additional 143 studies which did not meet our inclusion criteria, 133 full text articles were assessed for eligibility. 62 studies were excluded for reasons outlined in the Methods section. A total of 71 studies were included in our analysis

Figure 1 outlines the process of narrowing down the list of suitable papers for the review. Briefly, a total of 425 articles were identified using three databases, plus an additional seven from alternative sources. After the removal of 156 duplicate records, 276 records were screened for eligibility. Out of the screened records, 143 did not relate to the topic of this review and 62 other records were excluded following full‐text review due to reasons outlined in the Exclusion Criteria subsection. Overall, 71 publications that reported hazard ratios and 95% CI for primary and/or secondary endpoints in CRC or mCRC relative to NLR were found to be eligible for meta‐analysis.

### 3.1 Study characteristics

The main characteristics of included studies are outlined in Table 1. Briefly, 71 studies came from 13 countries, with the majority (*N* = 21) conducted on patient populations recruited in China. The studies were published between 2007 and 2020 (mean 2015) and accounted for a total of 32,788 patients, with individual study patient numbers ranging from 33 to 3008 (median 220, interquartile range (IQR) = 408). In 67 studies that reported patient sex, 15,244 patients were male (57%) and 11,333 were female (43%). The mean/median follow‐up length was 45 months. Finally, the median NLR cut‐off was 3.12 (IQR = 2.35), which was determined by data‐driven methods, such as receiver operating characteristic (ROC) curve analysis, in 34 publications (48%).

**TABLE 1:** Characteristics of the 71 studies included in the analysis

Study	Country	*N*	Males	Age^a^	Follow‐up^b^	AJCC stage	NLR cut‐off	ROC	NOS
Absenger (2013)^45^	Austria	504	293	65	45	II, III	4	−	5
Balde (2017)^46^	China	170	102	57.7	21.14	I, II, III, IV	3.5	+	4
Carruthers (2012)^47^	UK	115	75	63.8	37.1	I, II, III, IV	5	−	4
Cha (1) (2019)^48^	Korea	137	85	NR	67.8	III	3	−	6
Cha (2) (2019)^49^	Korea	131	86	59	73.3	II, III	3	−	5
Chan (2017)^50^	Australia	1623	801	NR	52	I, II, III	3.19	+	4
Chen (2015)^51^	USA	166	96	57	NR	IV	5	−	4
Chiang (2012)^52^	China	3008	NR	63	96.2	I, II, III	3	+	7
Choi (2014)^53^	Korea	105	63	63	44	I, II, III, IV	3	−	4
Choi (2015)^54^	Canada	549	296	68.7	48	I, II, III	2.6	+	6
Chua (2011)^55^	Australia	171	110	61	NR	IV	5	−	4
Clarke (2020)^56^	Australia	128	58	64	NR	IV	5	−	4
Climent (2019)^57^	Ireland	566	260	69.9	60	I, II, III	5	−	8
Dell’Aquila (2018)^58^	Italy	413	244	61	48.1	IV	3	+	5
Dimitrou (2018)^59^	Greece	296	182	72	NR	I, II, III	4.7	+	6
Ding (2010)^60^	China	141	78	61	58	II	4	+	4
Dudani (2019)^34^ ^,^ *	Canada	1237	858	62	71	II, III	4	−	7
Dupré (2019)^61^	UK	343	236	65.8	49	IV	2.6	+	5
East (2014)^62^	Ireland	50	30	79.6	42	I, II, III, IV	3.4	+	6
Feliciano (2017)^63^	USA	2470	1251	62.9	72	I, II, III	3	−	7
Galizia (2015)^64^	Italy	276	165	NR	NR	I, II	2.36	+	5
Ghanim (2015)^65^	Austria	52	31	62.7	NR	IV	4	−	5
Giakoustidis (2015)^66^	UK	169	104	NR	34.6	IV	2.5	+	6
Guthrie (2013)^67^	UK	206	120	NR	36	I, II, III, IV	5	−	5
Hachiya (2018)^68^	Japan	941	581	68.5	18.4	I, II, III, IV	2.9	+	5
He (2013)^69^	China	243	155	56	21.87	IV	3	−	6
Halazun (2007)^70^	UK	440	289	64	24	IV	5	−	6
Hung (2011)^71^	China	1040	561	NR	74.5	II	5	−	8
Jeon (2019)^72^	Korea	140	93	62.5	37	I, II, III	2.66	+	5
Jiang (2019)^73^	China	102	72	NR	33.2	IV	3.285	+	6
Kaneko (2012)^74^	Japan	50	33	61	17	IV	4	−	4
Ke (2020)^75^	China	184	121	63.2	72.73	I, II, III	3.5	−	7
Kim (2017)^76^	Korea	1868	1072	65	46	I, II, III, IV	3	+	6
Kim (2019)^77^	Korea	161	104	63.3	54	I, II, III, IV	2.17	+	6
Kishi (2009)^78^	USA	290	193	57	29	IV	5	−	5
Kubo (2016)^79^	Japan	823	457	67.1	48.5	I, II, III, IV	2.1	+	5
Kwon (2012)^80^	Korea	200	123	64	33.6	I, II, III, IV	5	−	6
Leitch (2007)^81^	UK	149	81	NR	48	I, II, III, IV	5	−	4
Liu (2010)^82^	China	123	NR	61.28	NR	I, II, III, IV	2	−	6
Loupakis (2019)^83^	Italy	395	198	65	33.9	IV	3	−	4
Mallappa (2012)^84^	UK	297	157	70	40.2	I, II, III, IV	5	−	5
Mao (2018)^85^	China	183	123	NR	36.3	IV	2.3	+	4
Matsuda (2019)^86^	Japan	33	20	69	NR	IV	5	−	4
Mercier (2019)^87^	Canada	152	95	NR	NR	IV	5.62	+	5
Mizuno (2019)^88^	Japan	892	511	68.6	58.7	II, III	5.5	+	7
Nagasaki (2015)^89^	Japan	201	140	NR	51.2	II	3	−	4
Neal (2009)^90^	UK	181	106	60.7	36	IV	5	−	5
Neal (2015)^91^	UK	302	192	64.8	29.7	IV	5	−	5
Oh (2016)^92^	Korea	261	143	65	78	II	2.6	+	7
Passardi (2016)^93^	Italy	289	174	NR	36	I, II, III, IV	3	+	5
Peng (1) (2017)^94^	China	150	97	58	36	IV	4.63	+	5
Peng (2) (2017)^95^	China	274	156	55	46	III	2.05	+	5
Rashtak (2017)^96^	USA	1622	NR	67	NR	I, II, III	3	+	4
Renaud (2018)^97^	France	574	338	65	62	IV	4.05	+	6
Sevinc (2016)^98^	Turkey	347	136	65	29.8	I, II, III, IV	3	−	4
Shimura (2018)^99^	Japan	35	20	NR	NR	I, II, III	2.9	+	4
Son (2013)^100^	Korea	624	368	NR	42	I, II, III	5	−	7
Song (2015)^101^	Korea	177	83	52	3.1	IV	5	−	4
Song (2017)^102^	China	1744	982	62	45.5	I, II, III, IV	2	+	7
Sun (2014)^103^	China	255	135	59.47	NR	I, II, III	5	−	6
Tao (2018)^104^	China	153	81	62.31	60	II, III, IV	2.24	+	7
Ucar (2020)^105^	Turkey	308	192	56	21.8	IV	3	−	4
Wang (2020)^106^	China	48	25	55	10.3	IV	4.1	−	5
Wei (2017)^107^	China	569	307	63	52	I, II, III	1.975	+	6
Weiner (2018)^108^	USA	131	84	59.1	NR	IV	5	−	4
Yang (2017)^109^	China	95	58	56	40	IV	2.34	−	5
Yang (2019)^110^	China	220	87	57	23.9	III, IV	2.65	+	4
Yatabe (2020)^111^	Japan	733	463	66	47.9	I, II, III, IV	2.4	−	4
Ying (2014)^112^	China	205	144	NR	NR	I, II, III	3.12	+	6
Zhang (2019)^113^	China	1458	NR	NR	44.9	I, II, III, IV	2.07	+	6
Zhao (2017)^114^	China	100	70	60.5	45.5	II, III	2.25	+	4

Note

‘+’ in the ROC column mark studies that used data‐driven methods such as ROC curves to define NLR cut‐offs.

Abbreviations: AJCC, American Joint Committee on Cancer; *N*, number of subjects; NOS, Newcastle–Ottawa Quality Assessment Scale score; NR, not reported; ROC, Receiver operating characteristic.

^a^ Mean or median years.

^b^ In months.

* Provided univariate HR and CI upon request.

### 3.2 Primary meta‐analysis: High NLR is prognostic of poor clinical outcome

We assessed NLR as a prognostic factor by pooling summary statistics of individual studies. This was performed separately for both univariate statistics, where NLR is used as a single explanatory variable in isolation, and multivariate results, where certain other potentially confounding variables are included and adjusted for, leading to a theoretically more accurate representation of NLR as a prognostic factor independently from other recorded variables.

**FIGURE 2:**
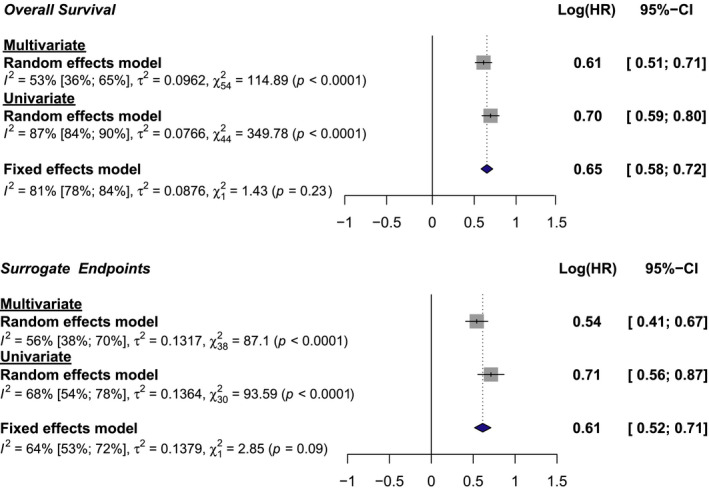
Forest plots of pooled hazard ratios (HR) and associated 95% confidence intervals (95%‐CI) of the effect of high versus low NLR for overall survival and surrogate endpoints in patients with colorectal cancer. Random effects models were used to pool HR in univariate and multivariate studies. Fixed effects models were used to compare univariate and multivariate pooled random effects natural log(HR)s. NLR, neutrophil‐to‐lymphocyte ratio

Of the 71 studies included, 45 reported univariate and 55 reported multivariate HR for OS, while 31 papers reported univariate and 39 reported multivariate HR for SE. High NLR was associated with significantly reduced OS, with a pooled effect size of HR = 2.01, 95% CI 1.81–2.21 in univariate and HR = 1.84, 95% CI 1.68–2.03 in multivariate analyses (*p* < 0.0001; Figure 2; Figure S1). Similarly, CRC and mCRC patients with high NLR had reduced SE compared to those with low NLR (HR = 2.04, 95% CI 1.75–2.37 for univariate; HR = 1.72, 95% CI 1.51–1.95 for multivariate data; *p* < 0.0001; Figure 2; Figure S2). Between‐study heterogeneity was lower in multivariate studies compared to univariate studies for both outcomes (OS: multivariate *I* ^2^ = 53%, univariate *I* ^2^ = 87%; SE: multivariate *I* ^2^ = 56%, univariate *I* ^2^ = 68%). Subgroup analyses between univariate and multivariate HR were used to assess confounding of NLR with other covariates adjusted for in our included studies. We found no significant differences between covariate‐adjusted and univariate data for either OS or SE (OS: mixed‐effects model, *χ*² = 1.43, *p* = 0.23, SE: mixed‐effects model, *χ*² = 2.85, *p* = 0.09; Figure 2). Overall, high pre‐intervention NLR is associated with poor clinical outcomes in patients with CRC.

### 3.3 Multivariate data characteristics: Multivariate models fail to adjust for well‐established covariates

To better understand the data and its potential confounding with particular variables, we investigated the covariates that multivariate models accounted for (Figures S3 and S4) in included publications. The median number of covariates used was 6 for OS (IQR = 5) and 7 for SE (IQR = 4), and while some papers only adjusted for a single factor, others included as many as 16 (Figures S3 and S4).

Due to the wide variety of factors used, we grouped the covariates into ‘Conventional’, ‘FBC‐derived’ and ‘Other’ (less common, usually study‐specific factors; see Figures S3 and S4). Briefly, individual studies included up to seven Conventional (median 2, IQR = 3 (OS) or 3.5 (SE)) or FBC‐derived factors (median 1, IQR = 1) and up to 10 Other factors (median 3, IQR = 2). Most studies (*N* = 53, 96% for OS, Figure S3; *N* = 35, 90% for SE, Figure S4) accounted for at least one type of conventional factor. Stage and age were the most popular conventional covariates, but even these were adjusted for by only about half of all papers, followed by sex and CEA. Similarly, only 51% of studies (*N* = 28 in OS, *N* = 20 in SE) accounted for any of the FBC‐derived factors, with PLR being the most popular covariate, followed by various other immune cell counts (e.g. leucocyte number, eosinophil count, basophil count), and LMR. All studies included in SE analysis accounted for at least one ‘Other’ type of factor, while only 49 (89%) did in OS.

Overall, covariates were found to be highly heterogeneous, with little consistency between studies. Importantly, only half of all papers included well‐established key factors outlined in current guidelines.

### 3.4 Subgroup analysis and meta‐regression: Studies with more patients looked at more covariates and reported lower hazard ratios for clinical outcomes

We next sought to investigate how certain study and patient characteristics may have been associated with heterogeneity in reported effect sizes. To this end, we performed meta‐regression and subgroup analyses for subsets of studies.

**FIGURE 3:**
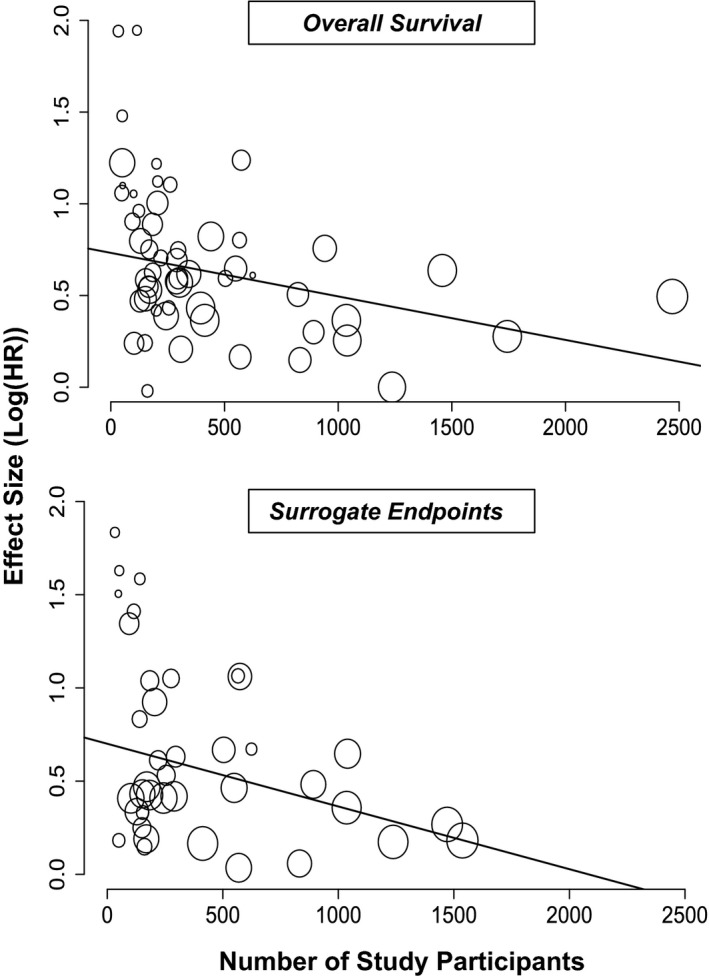
Association between study effect size and the number of participants. The circles indicate effect sizes (natural log of hazard ratios, log(HR)) of high versus low NLR on overall survival or surrogate endpoints in colorectal cancer patients and the number of participants in individual studies. The size of each circle is inversely proportional to the variance of the estimated treatment effect. The solid line represents the line of best fit

Mixed‐effects meta‐regression model was used to assess potential relationships between continuous variables (e.g. age) and effect size (Table 2). The number of patients in studies negatively correlated with effect size for both primary and secondary outcomes (OS: *β* = −0.0002, *p* = 0.0071; SE: *β* = −0.0003, *p* = 0.0203), indicating that as the number of patients included in study increased, the reported hazard ratio decreased (Figure 3). Interestingly, there were no significant relationships between other continuous variables and the effect size (Table 2).

**TABLE 2:** Meta‐regression analysis of continuous variables in overall survival (upper) and surrogate endpoints (lower)

Overall survival				
Covariate	Study *N*	*β*	*p*‐value	Significance
Patient number	55	−0.0002	0.0071	**
Age^a^	42	0.0206	0.0738	
Publication year	55	−0.0253	0.1122	
Follow‐up^b^	44	−0.0047	0.1602	
Percentage male	53	−0.0001	0.9766	
NOS score	55	−0.0679	0.1229	
Factors adjusted for	55	−0.0131	0.4224	
*N* Conventional factors	55	−0.0438	0.1653	
*N* FBC‐derived factors	55	−0.0047	0.8972	
NLR cut‐off	55	0.0333	0.4421	

Surrogate endpoints (disease‐, progression‐ and recurrence‐free survival)				
Covariate	Study *N*	*β*	*p*‐value	Significance
Patient number	39	−0.0003	0.0203	*
Age^a^	28	0.0064	0.7787	
Publication year	39	−0.0046	0.8431	
Follow‐up^b^	30	−0.0022	0.5310	
Percentage male	37	0.0049	0.1970	
NOS score	39	−0.0657	0.2624	
Factors adjusted for	39	−0.0369	0.0628	
*N* Conventional factors	39	−0.0376	0.2572	
*N* FBC‐derived factors	39	0.0574	0.8940	
NLR cut‐off	39	0.0871	0.1568	

Abbreviations: *β*, regression coefficient; FBC, full blood count; NLR, neutrophil‐to‐lymphocyte ratio; NOS, Newcastle‐Ottawa Scale.

^a^ Mean or median years.

^b^ In months.

**FIGURE 4:**
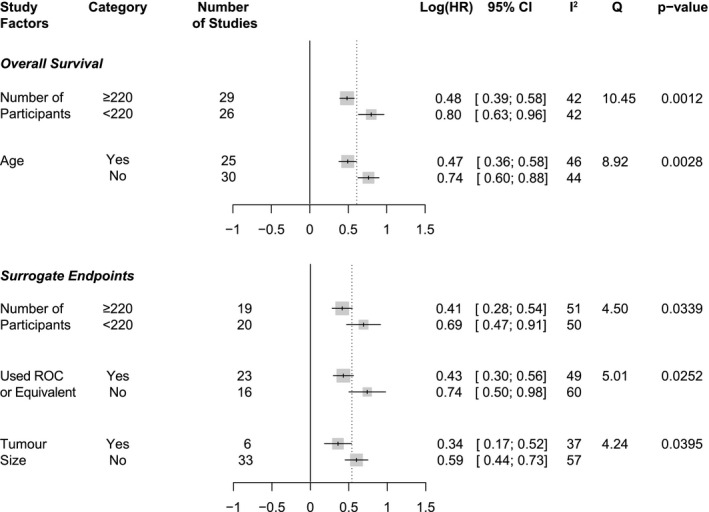
Subgroup analysis of categorical variables in multivariate studies. Forest plots representing the difference in pooled group effect size on overall or survival or surrogate endpoints based on study characteristics. Only statistically significant factors are presented; for the full dataset, see Table S1. Between‐groups analysis was carried out using fixed effects models. CI, confidence interval, ROC, Receiver Operating Characteristic curve

We next assessed the relationship between the categorical variables (e.g. geographical location) and effect size by performing subgroup analyses (statistically significant results are presented in Figure 4, complete dataset available in Table S1). In the subgroup analysis of OS factors, studies that had fewer than 220 participants (*p* = 0.0012) or did not adjust for age (*p* = 0.0028) reported a significantly higher HR (Figure 4). Interestingly, there were no significant relationships recorded for other factors, including stratification by AJCC stage. In the subgroup analysis of SE factors, studies that did not adjust for tumour size reported significantly larger HR (*p* = 0.0395; Figure 4). Additionally, studies that did not use data‐driven methods (e.g. ROC) to define NLR cut‐offs or that had fewer than 220 patients also reported significantly higher HR for SE (*p* = 0.0252 and 0.0339 respectively; Figure 4).

Several studies included covariates in multivariate analysis only if they were statistically significant in univariate analysis. However, a study with more participants and, consequently, more statistical power, is more likely to achieve significance for potential covariates in a univariate model. Indeed, studies with more than the median 220 participants adjusted for more covariates (OS: *t* = 2.553, *p* = 0.0136; SE: *t* = 2.578, *p* = 0.0141; Student's *t* test). Also, for OS, there was a positive correlation between studies with ≥220 participants and adjusting for age (Pearson's *Φ* = 0.2793, *p* = 0.0384). This could partly explain why studies that adjusted for age showed a significantly lower effect size in our subgroup analysis.

Overall, studies including more patients adjusted for more covariates and were associated with lower hazard for both overall survival and surrogate endpoints regardless of whether it was treated as a continuous or categorical variable, indicating the possibility of publication bias.

### 3.5 Publication bias

Due to the observation that study size is negatively correlated with effect size, we investigated the presence of publication bias using funnel plots. For both OS and SE, funnel plots revealed significant asymmetry—the majority of smaller multivariate studies positioned to the right of the larger studies, showing a bias towards reporting higher prognostic effect estimates (Egger's test *t* = 3.588, *p* = 0.0007 for OS; *t* = 5.774, *p* < 0.0001 for SE; Figure S5).

Duval & Tweedie's trim‐and‐fill method was used to detect and adjust for publication bias by imputing small, ‘missing’ studies that were unpublished, likely due to unfavourable results (Figure S5). The bias‐adjusted results reduced effect size estimates by ~15% to HR = 1.57 (95% CI 1.39–1.78) for OS and by ~25% to HR = 1.38 (95% CI 1.16–1.64) for SE, remaining significant. Between‐study heterogeneity increased to *I* ^2^ = 66.5% (57.1%–73.8%) for multivariate OS and *I* ^2^ = 69.5% (59.7%–77.0%) for multivariate SE. Overall, despite the publication bias, high NLR remains prognostic of poor clinical outcome.

## 4 DISCUSSION

Recent years have seen a great increase in the number of publications reporting associations between poor prognosis and NLR in CRC. In this work, we identified and performed meta‐analysis of 71 publications to assess the utility of NLR as a prognostic marker of CRC. We found that high pre‐treatment blood NLR is associated with poor clinical outcomes in terms of overall survival and surrogate endpoints in CRC patients.

Additionally, this work highlighted methodological limitations of prognostic marker research. An ever‐increasing number of papers are published every day, of these, however, many rely on limited patient cohorts and are consequently prone to ‘small‐study effects’. These may distort findings and complicate the systematic evaluation of prognostic value.

### 4.1 Problems in covariate selection for multivariate analyses in prognostic studies

Comparison of pooled univariate and multivariate hazard ratios revealed no significant differences in effect size (Figure 2). At first, this may suggest that NLR is an excellent CRC outcome predictor that is not affected by other variables in multivariate models. However, closer investigation of covariates included in multivariate models revealed considerable heterogeneity and a lack of consistency between studies. During data extraction, we found that many reports only included factors in their multivariate models if they were statistically significant in univariate analyses. Such an algorithmic approach is inappropriate, as it ignores the theoretical relevance of certain variables.^115^ It also means that in smaller studies with less statistical power, even well‐established prognostic factors may be left out. Indeed, studies with less than 220 participants adjusted for fewer covariates (OS: *t* = 2.553, *p* = 0.0136; SE: *t* = 2.578, *p* = 0.0141). Consequently, the extent of residual confounding cannot be reliably gauged. Because this is a common issue that plagues reports of prognostic markers, we would urge the authors of prognostic factor studies to consult the literature and incorporate certain covariates in line with current guidelines specific for the condition they are studying (such as those published by ESMO for CRC^11^, ^12^), regardless of their statistical significance in univariate models.

### 4.2 Small studies inflate effect size estimates

Apart from impacting covariate inclusion, study size also showed a significant inverse correlation with effect size in our meta‐regression analyses (Table 2; Figure 3). As expected, considerable publication bias was observed that was limited to small studies (Figure S5). Correction for this bias reduced our best estimated effect size by 15% and 25% for OS and SE to HR = 1.57 (95% CI 1.39–1.78) and 1.38 (95% CI 1.16–1.64) respectively. Thus, accounting for small study bias reduces effect size, although not to the extent seen in some other examples of single prognostic markers.^116^

### 4.3 Data dichotomisation is an avoidable source of bias

Data‐driven dichotomisation, the selection of an ‘optimal’ cut‐off point that yields the minimal *p*‐value, is a well‐known source of bias.^117^ This approach was highly prevalent in our studies, with about half of the included reports relying on it, resulting in cut‐offs ranging from 1.975 to 5.62. The other half, on the other hand, used previously reported NLR cut‐offs or population medians (most commonly 5 or 3). This highlights the clear lack of consensus that complicates analysis and introduces further bias, especially in smaller studies.^118^ Interestingly, in the selection of cut‐off values, no reference was made to what the normal range of NLR is in healthy individuals. A non‐exhaustive search for publications reporting these values returned average NLR values ranging from 1.65 to 2.15.^119^, ^120^, ^121^, ^122^, ^123^ Individual studies, however, had a wide range of NLR values: in Forget et al.’s^120^ report, for example NLR values ranged between 0.78 and 3.58 in a healthy, active Belgian population. Gathering information about NLR values in matched healthy populations could provide some insight into the CRC‐specific changes and guide a better‐informed dichotomisation approach.

Having considered the impact of dichotomisation, the authors question the clinical relevance and appropriateness of this strategy. While dichotomisation simplifies the analysis and data presentation, it also complicates interpretation. For instance Altman and Royston^124^ argue that data dichotomisation reduces statistical power, overestimates the effect size and introduces a systematic ascertainment bias that cannot be corrected for by meta‐analyses. Thus, there is a compelling argument to measure the prognostic value of NLR without categorisation and instead as a continuous variable. This reflects the nature of the relationship between a predictor and response and, importantly, is still easy to implement in a clinical setting as a decision‐aiding tool.

### 4.4 Recommendations for future prognostic reports

Overall, more work is required to establish a high‐quality link between FBCs such as NLR and clinical outcome. Future studies should pay more attention to the trends unfolding in their chosen area of interest, such as established cut‐offs. The authors should also be conscious about the limitations of their datasets, such as a low number of participants, and not allow this to guide their decision when it comes to the inclusion of established covariates. Riley et al.^125^ outline a number of guidelines to improve publishing standards and facilitate systematic reviews which could serve as a starting point for future prognostic factor reports, supplementing publishing standards such as the REMARK guidelines.^126^, ^127^

### 4.5 Limitations

One key limitation of this review is the lack of individual patient‐level data in the studies included. Only summary statistics were available for extraction and synthesised into this work. In the digital era, vast amount of health data is accumulated for clinical purposes with the potential to be repurposed, shared, combined and analysed for the public good.^128^ This is complicated by issues regarding confidentiality and consent, and resulted in the suspension of schemes as ambitious (and controversial) as the NHS’s care.data programme.^129^ Ethics boards generally waive the requirement for consent when it comes to retrospective analysis of patient databases, but this may not extend to the free sharing of datasets.

There is also the possibility of missed publications due to the highly focused search criteria (outlined in Section 2). Despite this limitation, this review is still the largest of its kind, since the highest number of papers included in other systematic reviews on colorectal cancer and NLR was 19.^33^

## 5 CONCLUSION

The information available from routine testing before cancer intervention, such as FBC, may provide valuable information regarding the patient outcome. There is a wealth of publications regarding the prognostic value of ratios of circulating immune cells in CRC. LMR,^130^ PLR^131^ and NLR have all been associated with the clinical outcome.

There is some cause for concern regarding the statistical rigour of cancer prognostic factor studies. Overall, reports showed no consistency in the way covariates were included in analyses. Most commonly accepted factors, such as age and tumour stage, were not included in the majority of analyses, particularly in smaller‐sized reports, due to the lack of statistical significance in univariate models. This highlights a need for a change in publishing standards when it comes to reporting prognostic markers. There is also need for large‐scale studies that assess prognostic factors accounting for conventional and newly proposed inflammation‐based markers.

Despite these shortcomings, using data from 71 publications accounting for 32,788 patients, we confirmed that high NLR is associated with poor patient outcome both in terms of overall survival (univariate: HR = 2.01, 95% CI 1.81–2.21; multivariate: HR = 1.84, 95% CI 1.68–2.03) and surrogate endpoints (univariate: HR = 2.04, 95% CI 1.75–2.37; multivariate: HR = 1.72, 95% CI 1.51–1.95). Correcting for the apparent publication bias in multivariate studies brought our best estimate for effect size down to HR = 1.57 (95% CI 1.39–1.78) for OS and to HR = 1.38 (95% CI 1.16–1.64) for SE. Based on these results, we believe that NLR could be used to highlight patients with tumour‐promoting inflammatory context. Furthermore, this comes at no additional cost, as blood tests are routinely carried out as part of or following a cancer diagnosis.

## CONFLICT OF INTEREST

The authors have declared no conflicts of interest.

## AUTHORS’ CONTRIBUTIONS

M.N. and T.S.M conceptualised the study. M.N. and A.K. reviewed titles, abstracts and full‐text papers for eligibility, extracted data and performed analysis. M.N. and A.K. wrote the manuscript with contributions from T.S.M.

## Supporting information

Fig S1

Fig S2

Fig S3

Fig S4

Fig S5

Table S1

## Data Availability

The data that support the findings of this study are available from the corresponding author upon reasonable request and are also presented in Figures [Link], [Link], [Link], [Link].
