# Supplementary figures and images for: The prognostic utility of pre‐treatment neutrophil‐to‐lymphocyte‐ratio (NLR) in colorectal cancer: A systematic review and meta‐analysis
Source: Cancer Med. 2021 Jul 26;10(17):5983–97. doi: 10.1002/cam4.4143 (PMC8419761; doi:10.1002/cam4.4143)

Overall Survival

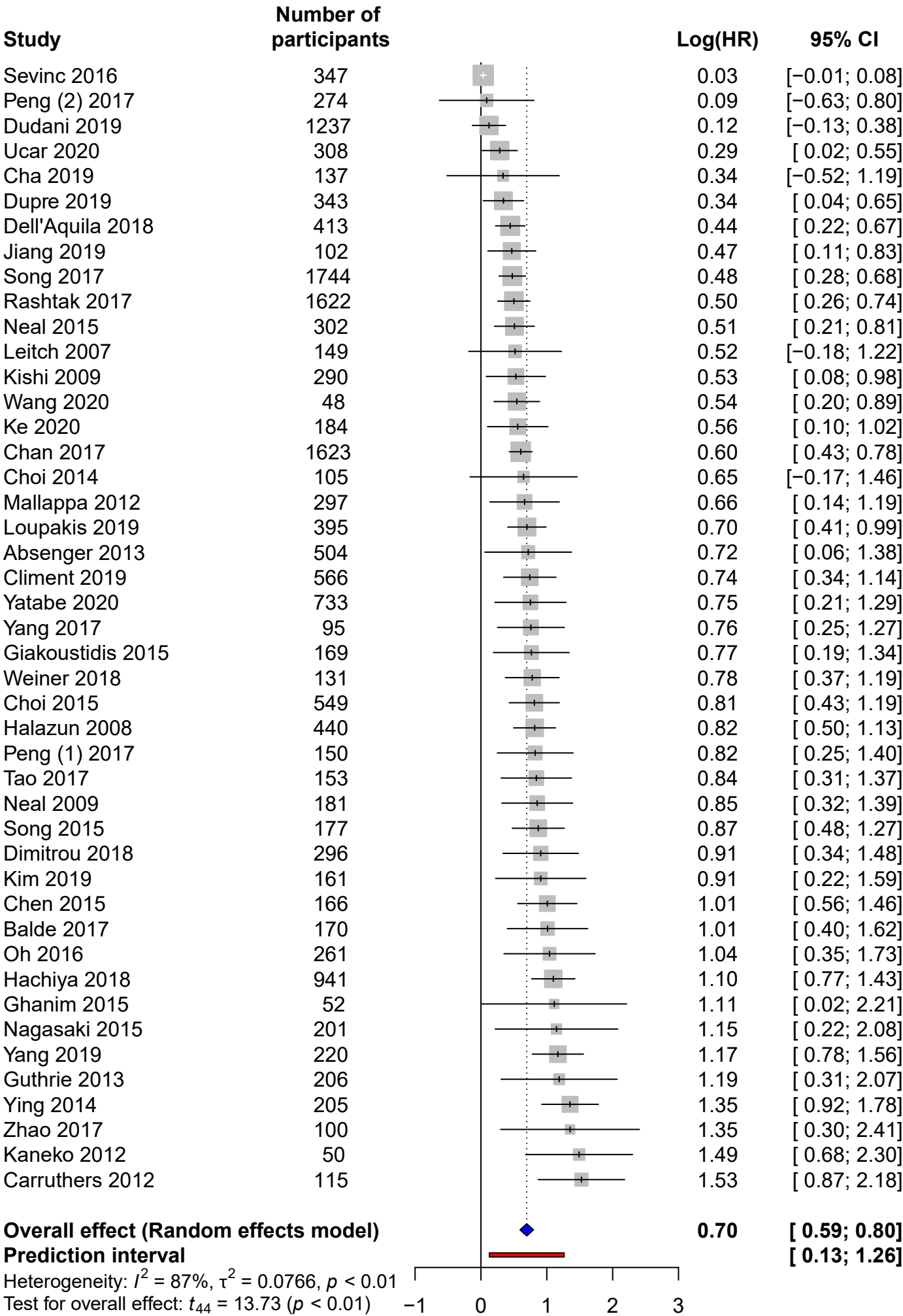

Supplement: Supplementary file 1 — Fig S1 [file CAM4-10-5983-s004.pdf]

Surrogate Endpoints

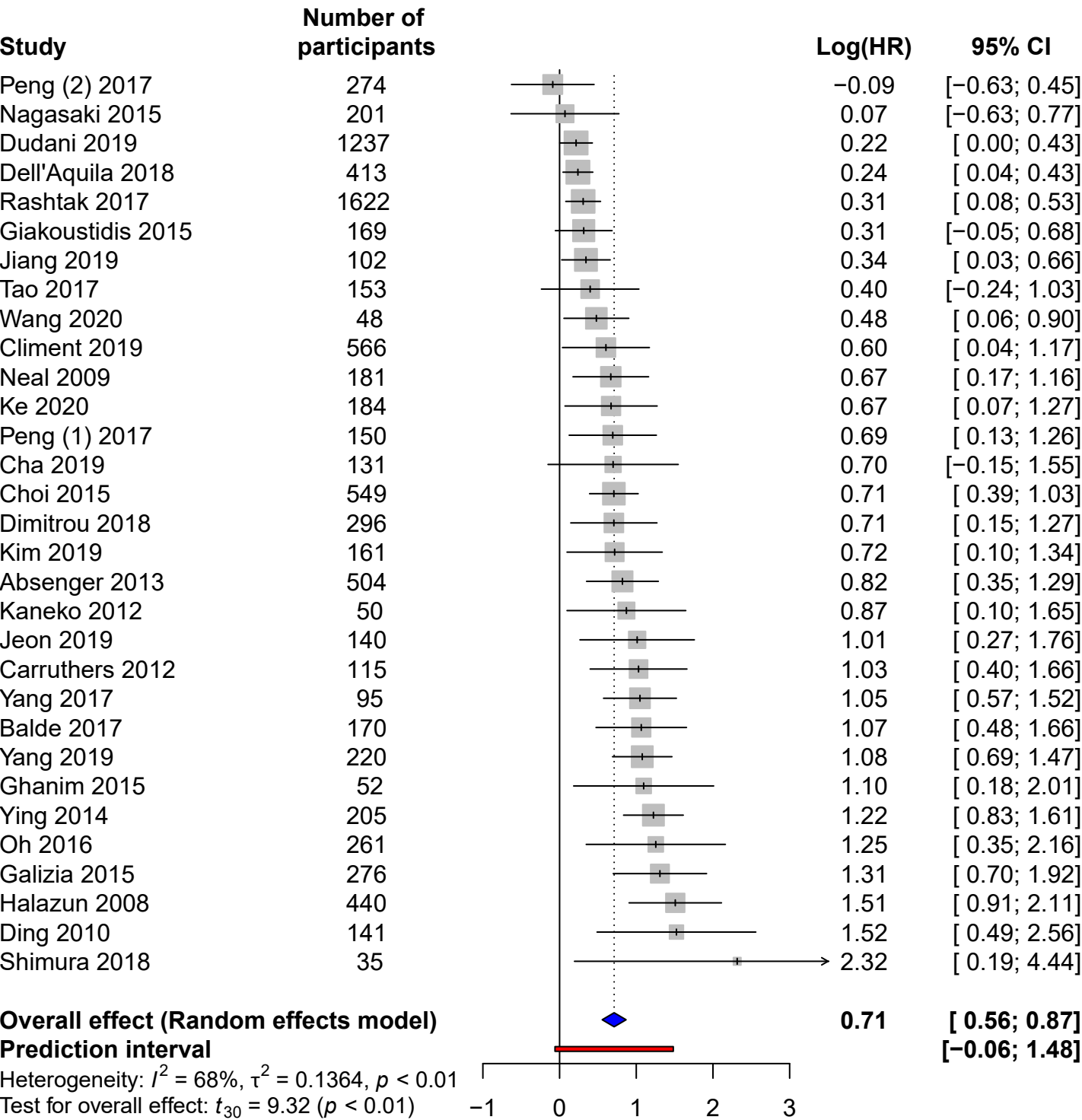

Supplement: Supplementary file 2 — Fig S2 [file CAM4-10-5983-s003.pdf]

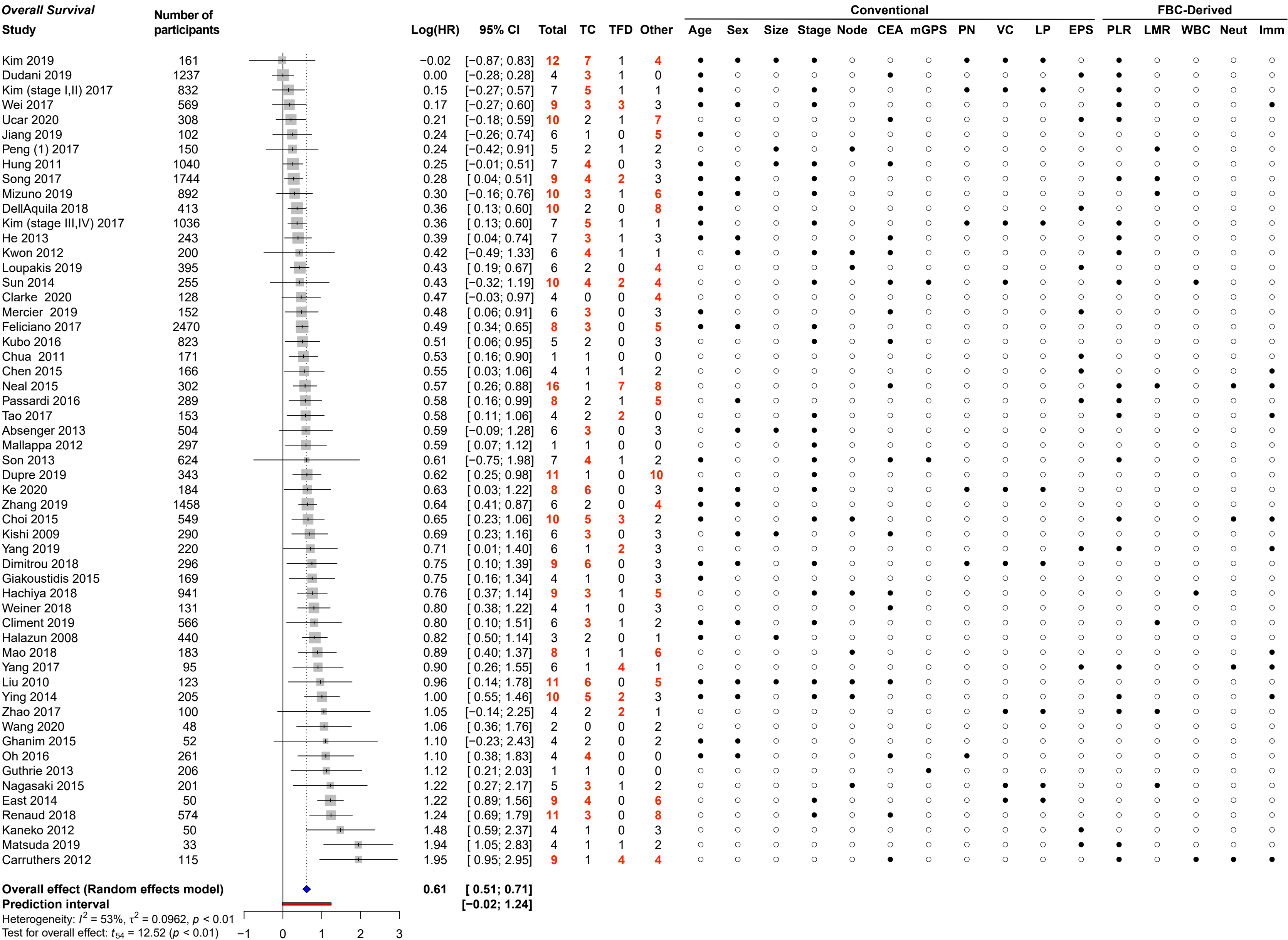

Supplement: Supplementary file 3 — Fig S3 [file CAM4-10-5983-s006.pdf]

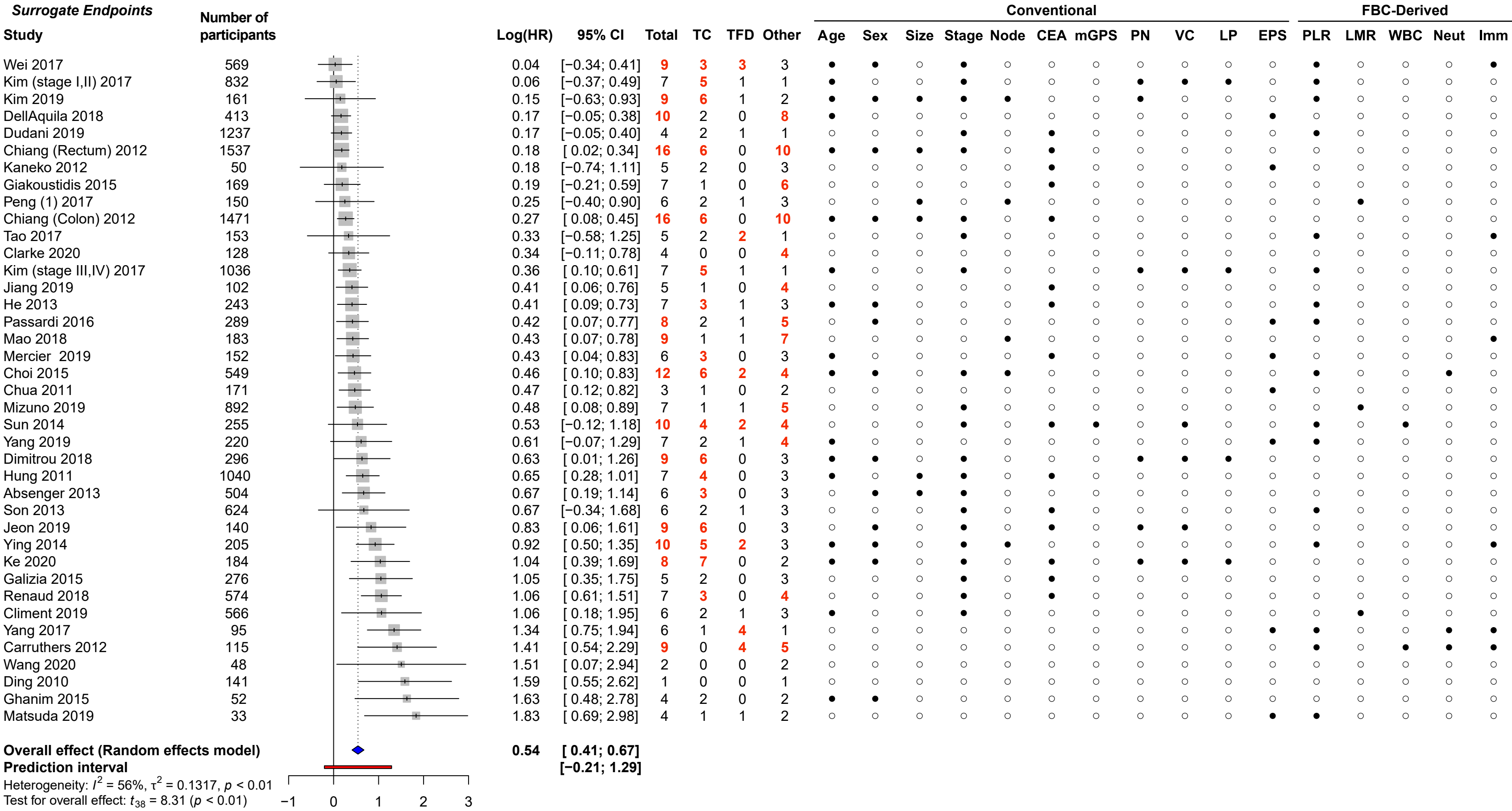

Supplement: Supplementary file 4 — Fig S4 [file CAM4-10-5983-s001.pdf]

Overall Survival

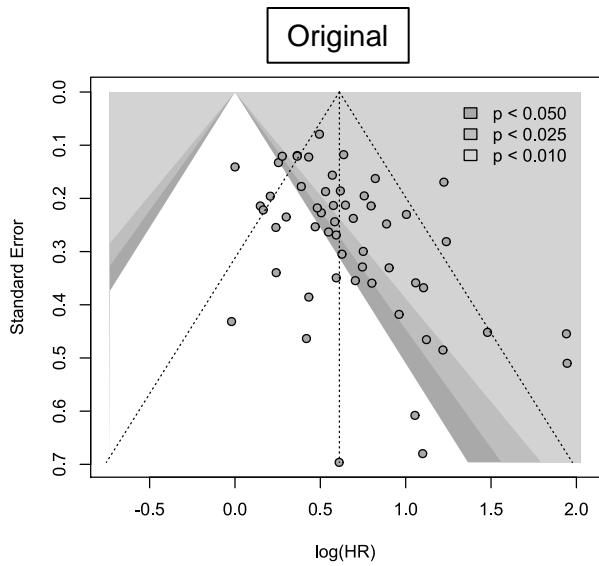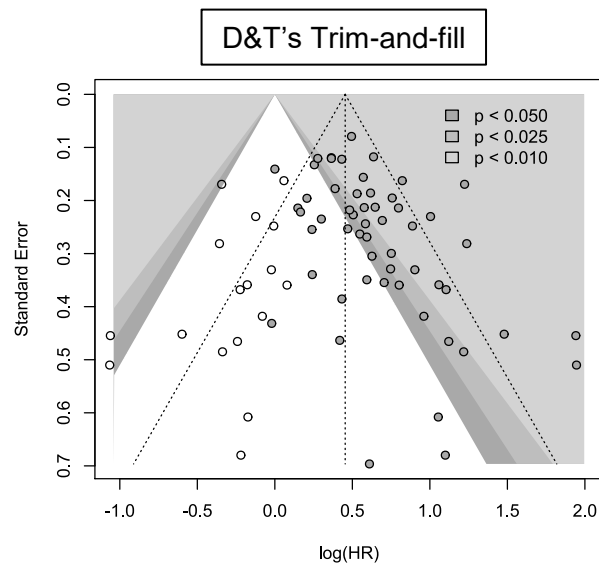

Surrogate Endpoints

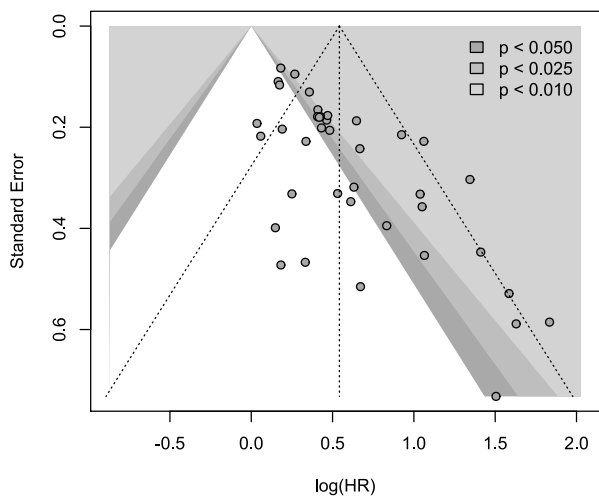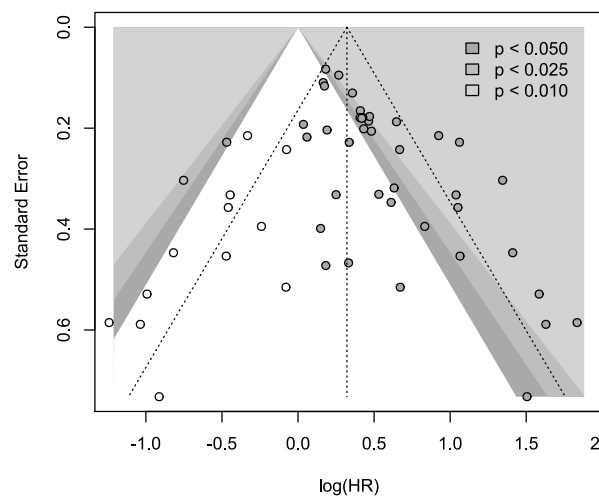

Supplement: Supplementary file 5 — Fig S5 [file CAM4-10-5983-s005.pdf]
