# Supplementary material for: The prognostic utility of pre‐treatment neutrophil‐to‐lymphocyte‐ratio (NLR) in colorectal cancer: A systematic review and meta‐analysis
Source: Cancer Med. 2021 Jul 26;10(17):5983–97. doi: 10.1002/cam4.4143 (PMC8419761; doi:10.1002/cam4.4143)
Supplement: Supplementary file 6 — Table S1 [file CAM4-10-5983-s002.docx]

## Supplementary Table S1. Subgroup analysis of categorical variables in multivariate studies. Statistically significant *p*-values are highlighted in bold. Within-groups analysis was carried out using random effects models; between-groups analysis for countries was carried out using random effects models, while all other factors were analysed using fixed (mixed) effects models. Groups with fewer than 4 studies per subgroup were not subject to between-groups analysis and are indicated with light grey font colour. HR: hazard ratio, CI: confidence interval, CEA: Carcinoembryonic antigen, DFS: disease-free survival, RFS: recurrence-free survival, TTR: time to recurrence, PFS: progression-free survival, PLR: Platelet/lymphocyte ratio, LMR: Lymphocyte/monocyte ratio, ECOG PS: Eastern Cooperative Oncology Group Performance Scale. ‘Immune cells’ factor refers to various immune cell numbers (e.g. leukocyte number, eosinophil count, basophil count).

| ***Overall survival*** | **Category** | ***N*** |  | **Within-groups Analysis** | | |  | **Heterogeneity** |  | **Between-groups Analysis** | |
| --- | --- | --- | --- | --- | --- | --- | --- | --- | --- | --- | --- |
| **Study Characteristics** |  |  |  | **HR** | **95% CI** | ***p*-value** |  | **I^2^ (%)** |  | **Q** | ***p*-value** |
| Country | UK | 7 |  | 2.23 | [1.56; 3.19] | <0.0001 |  | 28 |  | 6.19 | 0.1851 |
|  | USA | 4 |  | 1.77 | [1.43; 2.19] | <0.0001 |  | 0 |  |  |  |
|  | Japan | 6 |  | 2.54 | [1.34; 4.82] | 0.0001 |  | 67 |  |  |  |
|  | China | 17 |  | 1.73 | [1.48; 2.02] | <0.0001 |  | 39 |  |  |  |
|  | Korea | 6 |  | 1.48 | [1.01; 2.16] | 0.0441 |  | 17 |  |  |  |
| AJCC Stage | I-III | 17 |  | 1.65 | [1.40; 1.95] | <0.0001 |  | 50 |  | 2.14 | 0.3432 |
|  | I-IV | 15 |  | 1.94 | [1.59; 2.36] | <0.0001 |  | 62 |  |  |  |
|  | IV | 23 |  | 1.92 | [1.66; 2.22] | <0.0001 |  | 45 |  |  |  |
| Number of Participants | <220 | 26 |  | 2.22 | [1.88; 2.62] | <0.0001 |  | 42 |  | 10.45 | **0.0012** |
|  | ≥220 | 29 |  | 1.62 | [1.48; 1.78] | <0.0001 |  | 42 |  |  |  |
| Used ROC or | no | 29 |  | 1.92 | [1.66; 2.23] | <0.0001 |  | 53 |  | 0.56 | 0.4541 |
| Equivalent | yes | 26 |  | 1.79 | [1.58; 2.02] | <0.0001 |  | 55 |  |  |  |
| **Covariates Adjusted For** | |  |  |  |  |  |  |  |  |  |  |
| Age | no | 30 |  | 2.10 | [1.83; 2.40] | <0.0001 |  | 44 |  | 8.92 | **0.0028** |
|  | yes | 25 |  | 1.60 | [1.43; 1.79] | <0.0001 |  | 46 |  |  |  |
| Sex | no | 37 |  | 1.90 | [1.67; 2.16] | <0.0001 |  | 61 |  | 1.00 | 0.3175 |
|  | yes | 18 |  | 1.73 | [1.53; 1.97] | <0.0001 |  | 20 |  |  |  |
| Tumour Size | no | 48 |  | 1.87 | [1.68; 2.08] | <0.0001 |  | 55 |  | 0.62 | 0.4323 |
|  | yes | 7 |  | 1.68 | [1.32; 2.14] | <0.0001 |  | 47 |  |  |  |
| Tumour Stage | no | 30 |  | 1.95 | [1.68; 2.25] | <0.0001 |  | 55 |  | 1.21 | 0.2715 |
|  | yes | 25 |  | 1.75 | [1.55; 1.98] | <0.0001 |  | 53 |  |  |  |
| Node Involvement | no | 46 |  | 1.82 | [1.63; 2.03] | <0.0001 |  | 56 |  | 0.79 | 0.3734 |
|  | yes | 9 |  | 2.00 | [1.67; 2.41] | <0.0001 |  | 23 |  |  |  |
| CEA | no | 38 |  | 1.86 | [1.67; 2.08] | <0.0001 |  | 48 |  | 0.05 | 0.8164 |
|  | yes | 17 |  | 1.81 | [1.49; 2.20] | <0.0001 |  | 62 |  |  |  |
| PLR | no | 34 |  | 1.93 | [1.75; 2.13] | <0.0001 |  | 41 |  | 1.10 | 0.2945 |
|  | yes | 21 |  | 1.71 | [1.40; 2.09] | <0.0001 |  | 59 |  |  |  |
| LMR | no | 48 |  | 1.87 | [1.68; 2.07] | <0.0001 |  | 55 |  | 0.80 | 0.3716 |
|  | yes | 7 |  | 1.66 | [1.30; 2.11] | <0.0001 |  | 23 |  |  |  |
| Neutrophil Number | no | 51 |  | 1.81 | [1.64; 1.99] | <0.0001 |  | 53 |  | 1.18 | 0.2773 |
|  | yes | 4 |  | 2.46 | [1.42; 4.25] | 0.0013 |  | 58 |  |  |  |
| Immune Cells | no | 45 |  | 1.80 | [1.62; 2.00] | <0.0001 |  | 54 |  | 1.02 | 0.3114 |
|  | yes | 10 |  | 2.06 | [1.62; 2.61] | <0.0001 |  | 42 |  |  |  |
| Perineural Invasion | no | 49 |  | 1.87 | [1.69; 2.07] | <0.0001 |  | 54 |  | 1.00 | 0.3168 |
|  | yes | 6 |  | 1.60 | [1.19; 2.15] | 0.0020 |  | 35 |  |  |  |
| Vascular Invasion | no | 46 |  | 1.84 | [1.66; 2.04] | <0.0001 |  | 49 |  | 0.00 | 0.9890 |
|  | yes | 9 |  | 1.85 | [1.38; 2.47] | <0.0001 |  | 70 |  |  |  |
| Lymphatic Invasion | no | 47 |  | 1.84 | [1.66; 2.03] | <0.0001 |  | 48 |  | 0.02 | 0.8834 |
|  | yes | 8 |  | 1.88 | [1.36; 2.60] | 0.0001 |  | 73 |  |  |  |
| ECOG PS | no | 43 |  | 1.87 | [1.69; 2.07] | <0.0001 |  | 46 |  | 0.08 | 0.7757 |
|  | yes | 12 |  | 1.80 | [1.37; 2.35] | <0.0001 |  | 65 |  |  |  |

| ***Surrogate endpoints*** |  | | ***N*** |  | **Within-groups Analysis** | | |  | **Heterogeneity** |  | **Between-groups Analysis** | |  |
| --- | --- | --- | --- | --- | --- | --- | --- | --- | --- | --- | --- | --- | --- |
| **Study Characteristics** | **Category** | |  |  | **HR** | **95% CI** | ***p*-value** |  | **I^2^ (%)** |  | **Q** | ***p*-value** | |
| Country | UK | | 2 |  | 1.46 | [1.37; 1.56] | <0.0001 |  | 59 |  |  |  | |
|  | USA | | 0 |  | 1.65 | [1.20; 2.27] | 0.0095 |  | 6 |  |  |  | |
|  | Japan | | 3 |  | 1.65 | [1.46; 1.87] | <0.0001 |  | 51 |  |  |  | |
|  | China | | 16 |  | 2.70 | [1.45; 5.03] | 0.0018 |  | 76 |  | 1.77 | 0.1831 | |
|  | Korea | | 5 |  | 1.40 | [0.98; 1.99] | 0.0634 |  | 0 |  |  |  | |
| AJCC Stage | I-III | | 18 |  | 1.69 | [1.42; 2.01] | <0.0001 |  | 60 |  | 0.25 | 0.8827 | |
|  | I-IV | | 6 |  | 1.64 | [1.22; 2.21] | 0.0012 |  | 16 |  |  |  | |
|  | IV | | 15 |  | 1.80 | [1.40; 2.31] | <0.0001 |  | 62 |  |  |  | |
| Number of Participants | <220 | | 20 |  | 2.00 | [1.60; 2.49] | <0.0001 |  | 50 |  | 4.50 | **0.0339** | |
|  | ≥220 | | 19 |  | 1.51 | [1.33; 1.72] | <0.0001 |  | 51 |  |  |  | |
| Used ROC or | no | | 16 |  | 2.09 | [1.65; 2.66] | <0.0001 |  | 60 |  | 5.01 | **0.0252** | |
| Equivalent | yes | | 23 |  | 1.54 | [1.35; 1.75] | <0.0001 |  | 49 |  |  |  | |
| Outcome Measure | | DFS/RFS/TTR | 27 |  | 1.72 | [1.47; 2.00] | <0.0001 |  | 59 |  | 0.01 | 0.9402 | |
|  | | PFS | 12 |  | 1.74 | [1.35; 2.23] | <0.0001 |  | 53 |  |  |  | |
| **Covariates Adjusted For** | | |  |  |  |  |  |  |  |  |  |  | |
| Age | no | | 22 |  | 1.88 | [1.56; 2.27] | <0.0001 |  | 54 |  | 2.26 | 0.1331 | |
|  | yes | | 17 |  | 1.55 | [1.32; 1.83] | <0.0001 |  | 54 |  |  |  | |
| Sex | no | | 26 |  | 1.77 | [1.49; 2.09] | <0.0001 |  | 56 |  | 0.30 | 0.5825 | |
|  | yes | | 13 |  | 1.64 | [1.35; 2.00] | <0.0001 |  | 58 |  |  |  | |
| Tumour Size | no | | 33 |  | 1.80 | [1.55; 2.08] | <0.0001 |  | 57 |  | 4.24 | **0.0395** | |
|  | yes | | 6 |  | 1.41 | [1.18; 1.68] | 0.0001 |  | 37 |  |  |  | |
| Tumour Stage | no | | 18 |  | 1.85 | [1.46; 2.35] | <0.0001 |  | 56 |  | 0.78 | 0.3764 | |
|  | yes | | 21 |  | 1.63 | [1.41; 1.89] | <0.0001 |  | 58 |  |  |  | |
| Node Involvement | no | | 34 |  | 1.75 | [1.51; 2.02] | <0.0001 |  | 59 |  | 0.17 | 0.6837 | |
|  | yes | | 5 |  | 1.65 | [1.28; 2.11] | 0.0001 |  | 26 |  |  |  | |
| CEA | no | | 24 |  | 1.81 | [1.50; 2.19] | <0.0001 |  | 57 |  | 0.97 | 0.3238 | |
|  | yes | | 15 |  | 1.60 | [1.36; 1.87] | <0.0001 |  | 55 |  |  |  | |
| PLR | no | | 23 |  | 1.74 | [1.49; 2.03] | <0.0001 |  | 55 |  | 0.03 | 0.8625 | |
|  | yes | | 16 |  | 1.69 | [1.35; 2.12] | <0.0001 |  | 61 |  |  |  | |
| LMR | no | | 36 |  | 1.46 | [1.37; 1.56] | <0.0001 |  | 59 |  |  |  | |
|  | yes | | 3 |  | 1.65 | [1.20; 2.27] | 0.0095 |  | 6 |  |  |  | |
| Neutrophil Number | no | | 36 |  | 1.65 | [1.46; 1.87] | <0.0001 |  | 51 |  |  |  | |
|  | yes | | 3 |  | 2.70 | [1.45; 5.03] | 0.0018 |  | 76 |  |  |  | |
| Immune Cells | no | | 33 |  | 1.67 | [1.47; 1.89] | <0.0001 |  | 48 |  | 0.68 | 0.4084 | |
|  | yes | | 6 |  | 2.03 | [1.30; 3.17] | 0.0019 |  | 77 |  |  |  | |
| Perineural Invasion | no | | 33 |  | 1.75 | [1.51; 2.02] | <0.0001 |  | 59 |  | 0.31 | 0.5755 | |
|  | yes | | 6 |  | 1.59 | [1.18; 2.14] | 0.0023 |  | 40 |  |  |  | |
| Vascular Invasion | no | | 33 |  | 1.73 | [1.50; 2.01] | <0.0001 |  | 59 |  | 0.11 | 0.7431 | |
|  | yes | | 6 |  | 1.65 | [1.25; 2.17] | 0.0004 |  | 38 |  |  |  | |
| Lymphatic Invasion | no | | 35 |  | 1.74 | [1.51; 2.00] | <0.0001 |  | 58 |  | 0.18 | 0.6671 | |
|  | yes | | 4 |  | 1.59 | [1.08; 2.34] | 0.0194 |  | 56 |  |  |  | |
| ECOG PS | no | | 31 |  | 1.70 | [1.48; 1.95] | <0.0001 |  | 55 |  | 0.12 | 0.7306 | |
|  | yes | | 8 |  | 1.81 | [1.28; 2.58] | 0.0009 |  | 67 |  |  |  | |
